# Non-suicidal self-injury: A bibliometrics study and visualization analysis from 2002 to 2022

**DOI:** 10.3389/fpsyt.2023.1019225

**Published:** 2023-02-09

**Authors:** Xiangli Dong, Yuchen Zou, Qing Zou, Na Zhao, Shilin Li, Guanxiu Liu, Maorong Hu, Weiming Sun

**Affiliations:** ^1^Department of Psychosomatic Medicine, The Second Affiliated Hospital of Nanchang University, Nanchang, China; ^2^Jiangxi Medical College, Nanchang University, Nanchang, China; ^3^Department of Rehabilitation Medicine, The First Affiliated Hospital of Nanchang University, Nanchang, China; ^4^Department of Psychosomatic Medicine, The First Affiliated Hospital of Nanchang University, Nanchang, China; ^5^Department of Rehabilitation Medicine, Jiangxi Provincial People’s Hospital, Nanchang, China; ^6^Department of Rehabilitation Medicine, Zhujiang Hospital of Southern Medical University, Guangzhou, China

**Keywords:** non-suicidal self-injury, bibliometric analysis, NSSI, WoSCC, CiteSpace, VOSviewer

## Abstract

**Objective:**

To overview the research actuality and offer the hotspots and cutting-edge issues in the field of Non-suicidal Self-injury (NSSI) by using bibliometric analysis.

**Materials and methods:**

Publications related to NSSI from 2002 to 2022 were extracted from the Web of Science Core Collection (WoSCC) database. CiteSpace V 6.1.R2 and VOSviewer 1.6.18 were used to visually analyzed institutions, countries, journals, authors, references, and keywords in research on NSSI.

**Results:**

A total of 799 studies about NSSI were analyzed *via* CiteSpace and VOSviewer. The number of annual publications related to NSSI is fluctuating growth. The USA and Harvard University are the most productive country and institutions. In the case of journals and co-cited journals, Psychiatry Research are the most productive journal and also ranked highest among co-cited journals. Furthermore, Michael Kaess has published the most publications, and Matthew K. Nock is the most cited author. An article published by Swannell SV et al. shows the highest citation counts. After analysis, the most common keywords are harm, adolescents and prevalence. The gender difference, diagnosis, and dysregulation are frontier areas of NSSI research.

**Conclusion:**

This study analyzed the research of NSSI from multiple perspectives, and provides valuable information for researchers to capture the current status, hot spots, and frontier trends of NSSI.

## 1. Introduction

Non-suicidal Self-injury (NSSI) is described as deliberate, direct destruction or alteration of body tissue without conscious suicidal intent (1). This includes any behave that maybe cause harm but not death to oneself, such as cutting, burning, scratching, and impingement (2). A study on NSSI have pointed out that an increasing trend globally, including in developing countries and it is almost equally prevalent in both developing and developed countries (3). A large number of studies has confirmed that NSSI is motivated by intrapersonal/self-regulating and interpersonal/social functions (3). Even people who show only one or two acts of NSSI admit at least one function for their act, and the intrapersonal function is the most common (4). NSSI has become a common mental health threat among people, the prevalence of NSSI is 46.5% in adolescents and 23% in young adults (5). There has high number of clinical assessment instruments and diversity of evaluations about NSSI research, the most frequent instruments of NSSI are structured interviews and their indicators were related to NSSI function and topography (6).

Many patients with non-suicidal suicide don’t take it seriously without treatment and eventually evolve into other psychiatric disorders such as borderline personality disorders (BPD), anxiety disorders, and affective disorders (7). The therapies of NSSI that were identified as effective were cognitive behavioral therapy, dialectic behavioral therapy for adolescents (DBT-A), and mentalization-based therapy for adolescents (MBT-A) (8). Furthermore, in recent study the cognitive behavior hypnotherapy was found to be effective in treating the non-suicidal self-injury condition (9).

Bibliometrics is a mathematical and statistical method that analyzes and observes research trends and is widely applied in many fields (10). It plays an important role in developing guidelines and understanding research hotspots. We can quickly clarify the structure of literature, analyze the development process, and capture the research hotspots of research fields by using bibliometrics analysis (11). This study aimed to use CiteSpace and VOSviewer scientific softwares to analyze literatures related to NSSI over the past 20 years and explore its research hotspots and development trends, and also to provide a thinking direction for future research.

## 2. Materials and methods

### 2.1. Data sources

**FIGURE 1:**
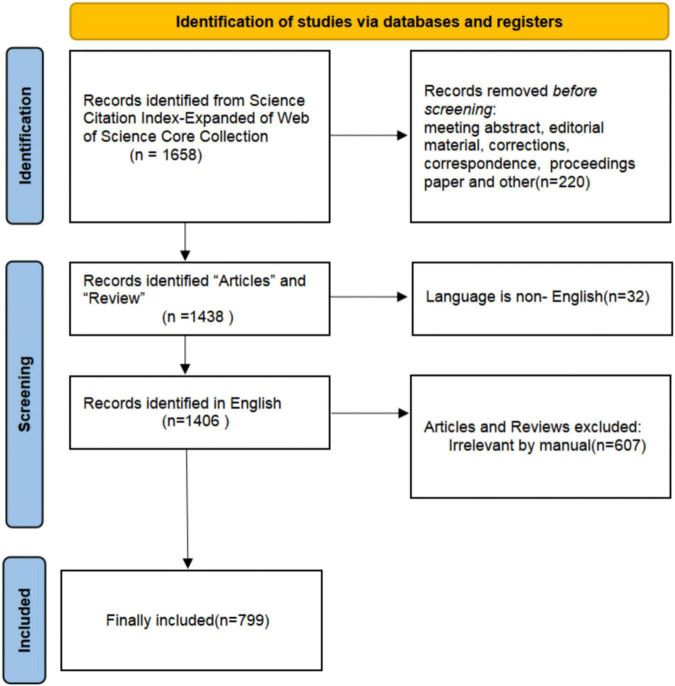
Flow chart of non-suicidal self-injury (NSSI) studies inclusion.

The retrieval data were extracted from the Science Citation Index-Expanded of Web of Science Core Collection (WoSCC). The WoSCC is the most frequently used and acceptable database for scientific or bibliometric studies. It contains nearly 9,000 of the world’s most prestigious high-impact journals and more than 12,000 academic conferences (12), so we selected it to perform the search. Relevant publications were downloaded within 1 day on December 4, 2022. The process of data extraction was presented in Figure 1. The search strategy was [TI = (non-suicidal self-injur* OR non-suicidal self-injur* OR non-suicidal self-harm* OR “NSSI”) OR AK = (non-suicidal self-injur* OR non-suicidal self-injur* OR non-suicidal self-harm* OR “NSSI”)]. To capture as many data sources as possible, the wildcard character (*) that could be substituted for any other characters and allows variable endings of keywords was used (13). For example, “non-suicidal self-injur*” would also return the terms of “non-suicidal self-injury” and “non-suicidal self-injurious.” The dates of the search restricted from January 1, 2002, to December 4, 2022, resulting in 1,658 records. This was followed by 220 unrelated document types, including conference abstracts, editorial material, corrections, correspondence, retractions, and conference proceedings, and 1,438 results were remaining. Then, restricting language as English there left 1,406 results. Next, two independent investigators reviewed the titles and abstracts and excluded articles that were not associated with NSSI, eventually 799 publications were remained.

### 2.2. Analysis tool

All search datas retrieved WOSCC were converted to VOSviewer and CiteSpace to perform visual analysis.

VOSviewer is a computer program developed by Van Eck and Waltman at Leiden University in the Netherlands in 2009 that is suitable for constructing complex networks using large-scale data (14). It provides visual analysis and creates maps based on network data. It can construct network diagrams of academic publications, scientific journals, authors, research institutions, countries, and keywords. The items in these networks can be connected by co-citation links, co-occurrence, citation, and bibliographic coupling (15). Each node of the network maps represented elements such as country, institution, or keyword, the size and color of the nodes reflected frequency and cluster of elements, respectively (16). The links between nodes represented the co-authorship, co-citation or co-occurrence associations between nodes and the color of the nodes and lines indicated different clusters or corresponding average appearing year (AAY) (17). We use VOSviewer to analyze institution, co-cited journals, authors, co-cited authors and keywords. VOSviewer software parameter settings: Normalization Method: association strength; the minimum publication thresholds for institutions and authors are all 5, respectively; the minimum citation thresholds for journals, authors, and references are 100, 100, and 50, respectively; and the minimum keyword occurrence threshold is 15 (18).

CiteSpace is created by Dr. Chaomei Chen (School of Information Science and Technology, Drexel University, Philadelphia, PA, USA) and his team in 2004 (11). Burst detection, betweenness centrality, and heterogeneous networks are three central concepts of CiteSpace, which help to quantitatively and visually summarize previous research, and identify trends and key change nodes in a timely manner. We used CiteSpace to analyze institution, countries, dual-map overlay of journals and strongest citation bursts of references and keywords. We downloaded the records retrieved by WoSCC, and then converted these data into plain text format for export, including complete records and references, which was named download_ XXX. txt, and finally imported into citespace (V.6.1.R2) for bibliometric and visual analysis (19). The specific parameters used in CiteSpace were set as follows: time span (from January 1, 2004 to December 31, 2022, year per slice = 1), top N (50), term source (title, abstract, author keywords, and keywords plus), node types (author, institution, country, keywords, cited reference, cited author, and cited journal), network clipping method (pathfinder) (20). The remaining settings maintain the software default. Different nodes in a map represented indexes including a country and institution, or journal. The size of the nodes spoke on behalf of the centrality of publications or frequency, and a node with large size typically indicated high occurrence or citation frequency as a pivotal point, meanwhile, the links between nodes showed the network of cooperation, collaboration, or co-citation, and the color of nodes and links indicated different clusters (21).

## 3. Results

### 3.1. Analysis of publication years

**FIGURE 2:**
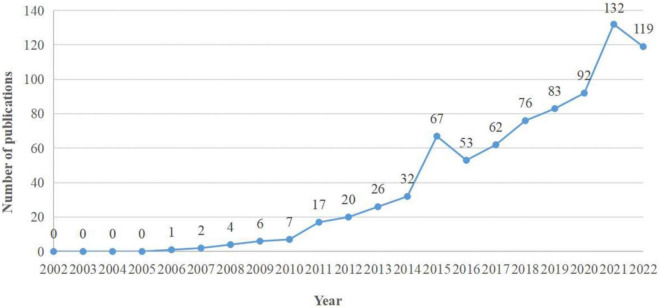
Trends in the number of publications on NSSI from 2002 to 2022.

The number of publications per specific period reflects the development trend of research in this field (Figure 2; 14). As shown in Figure 2, From 2002 to 2010, publication outputs during this period were low, the research on NSSI had a slow development speed. Since 2011, the number of publications has gradually increased, reaching 132 publications output in 2021, indicating that NSSI is receiving increasing attention.

### 3.2. Analysis of institutions

**FIGURE 3:**
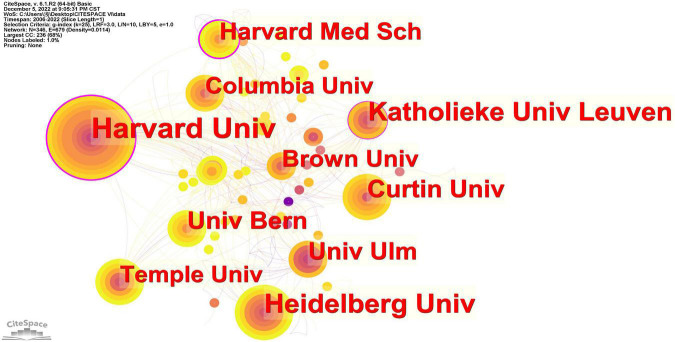
An institution cooperation map related to non-suicidal self-injury (NSSI) research by CiteSpace. The larger circle is, the more publications output of institution is; the links between nodes showed the network of cooperation, the denser the link, the more cooperation of institution is.

**FIGURE 4:**
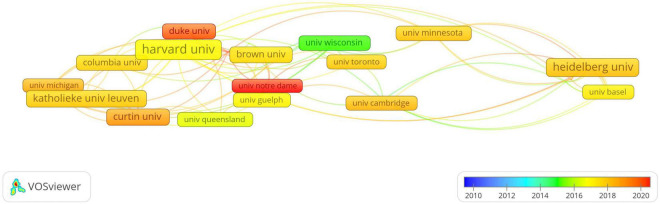
The overlay visualization of institutions generated by VOSviewer. The color of the nodes and lines indicated corresponding average appearing year of institution.

A total of 346 institutions involved in the publications about NSSI and the top 10 productive institutions are listed in Table 1. Harvard University (*n* = 40) has the most publications, followed by Heidelberg University (*n* = 31) and Katholieke University Leuven (*n* = 27). The network map of institution cooperation was generated by CiteSpace (Figure 3), in which each node represents an institution and the size of the node indicates the number of publications by the institution. The bigger the size of nodes is, the more publication output of institution is. Besides, the purple outer ring of the circle is centrality, the wider the circle, the higher is the centrality (22). It can be seen that there were relatively few collaborations among institutions from different countries, only three institutions including Harvard University (centrality: 0.34), Harvard Medical School (centrality:0.26) and Katholieke University Leuven (centrality:0.14) had the value of centrality more than 0.1. The institution co-authorship analysis was generated by VOSviewer (Figure 4). According to the color gradient in the lower right corner, these institutions such as Duke University and University of Notre Dame were given a red color with the larger AAY values, University of Wisconsin System is given a green color with the smaller AAY values (17).

**TABLE 1:** The top 10 institutions related to non-suicidal self-injury (NSSI).

Ranking	Institution	Publications	Percent	Centrality
1	Harvard University	40	16.81%	0.34
2	Heidelberg University	31	13.03%	0.05
3	Katholieke Universiteit Leuven	27	11.35%	0.14
4	Curtin University	22	9.24%	0.02
5	University of Bern	22	9.24%	0.03
6	Ulm University	21	8.82%	0.09
7	Harvard Medical School	20	8.40%	0.26
8	Brown University	19	7.98%	0.09
9	Temple University	18	7.56%	0.06
10	Columbia University	18	7.56%	0.09

### 3.3. Analysis of countries and funding agencies

**FIGURE 5:**
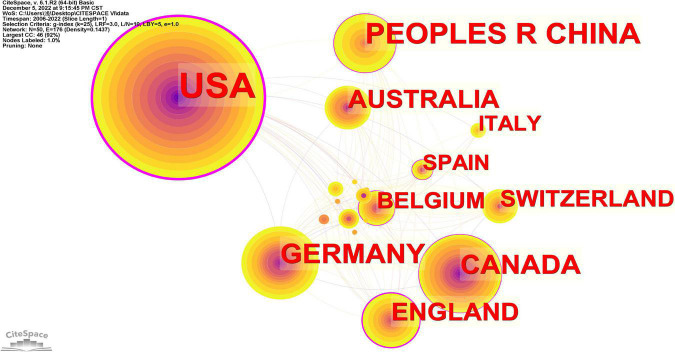
A country cooperation map related to non-suicidal self-injury (NSSI) by CiteSpace. The large node is, the more publications output of countries is.

According to statistics, a total of 799 articles related to NSSI were published in 50 countries. The top 10 countries are listed in Table 2. The United States had the most publications (*n* = 330), followed by China (*n* = 109) and Germany (*n* = 84). This phenomenon may be related to the population of the countries. The larger the population, the greater the number of psychological problems associated with NSSI, which can arouse the attention of clinical workers and researchers. The country cooperation map was conducted CiteSpace (Figure 5), in which each node represents a country, and the size of the node is proportional to the number of articles published by country. Links between nodes signify relationships of collaboration or co-occurrence or co-citation among countries (23). The figure shows the close cooperation between the USA and other countries including Belgium, Australia, and Germany etc. Besides, The USA leads in both single-country and multi-country publications among different countries. It’s worth noting that Italy’s centrality is 0, which indicates Italy has relatively few collaborations with other countries. In Table 3, top 10 funding sources in the field of NSSI research are listed. summarizes the data of the top 10 most frequent funding sources in this field. Obviously, the United States has a large percentage, with five funding agencies in the United States. The USA also published the most relevant articles, it is apparent that the wealth of different countries can influence research in certain degree.

**TABLE 2:** The top 10 countries related to non-suicidal self-injury (NSSI).

Ranking	Country	Publications	Percent
1	USA	330	39.52%
2	China	109	13.05%
3	Germany	84	10.06%
4	Canada	75	8.98%
5	Australia	55	6.59%
6	England	53	6.35%
7	Switzerland	37	4.43%
8	Belgium	35	4.19%
9	Spain	29	3.47%
10	Italy	28	3.35%

**TABLE 3:** The top 10 funding agencies.

Ranking	Funding agencies	Countries	Percent
1	United States Department of Health Human Services	USA	18.273%
2	National Institutes of Health	USA	17.772%
3	National Institute of Mental Health	USA	14.143%
4	National Natural Science Foundation of China	China	6.508%
5	European Commission	Europe	5.006%
6	Canadian Institutes of Health Research	Canada	2.003%
7	American Foundation For Suicide Prevention	USA	1.752%
8	National Health and Medical Research Council of Australia	Australia	1.752%
9	National Science Foundation	USA	1.627%
10	Swedish Research Council	Sweden	1.627%

### 3.4. Analysis of journals and cited journals

**FIGURE 6:**
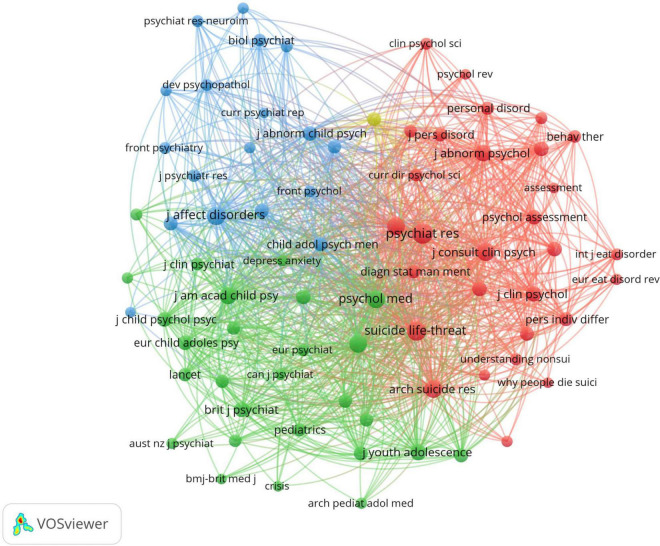
Network visualization map of journal co-citation analysis generated by VOSviewer. Each node represents a co-citation journal and each color represents one cluster, the links between nodes represents co-citation of journals.

To find the core and authoritative journals in the field of NSSI, we conducted the analysis of literature sources (24). The top 10 journals are shown in Table 4. The first was Psychiatry Research (*n* = 105), followed by journal of affective disorders (*n* = 63) and frontiers in psychiatry (*n* = 57). The highest impact factor journal was the Psychiatry Research (IF = 11.225, JCR = Q1), and the average of 10 journals’ impact factor was approximately 6.44. The network visualization map of journal co-citation analysis was generated by VOSviewer (Figure 6). Only journals with a minimum of 100 citations were visualized and 75 journals satisfying the criteria, the most frequency co-citations is Psychiatry Research, followed by Suicide And Life-threatening Behavior and Journal Of Affective Disorders.

**TABLE 4:** The top 10 journals with the highest frequency of non-suicidal self-injury (NSSI).

Ranking	Journal	Frequency (2020)	IF (2021)	JCR
1	Psychiatry Research	105	11.225	Q1
2	Journal of Affective Disorders	63	6.533	Q1
3	Frontiers in Psychiatry	57	5.435	Q2
4	Comprehensive Psychiatry	32	7.211	Q1
5	Child and Adolescent Psychiatry and Mental Health	31	7.494	Q1
6	BMC Psychiatry	30	4.144	Q2
7	International Journal of Environmental Research and Public Health	23	4.614	Q2
8	Psychological Medicine	17	10.592	Q1
9	Journal of Nervous and Mental Disease	15	1.899	Q4
10	Journal of Psychiatric Research	15	5.25	Q2

**FIGURE 7:**
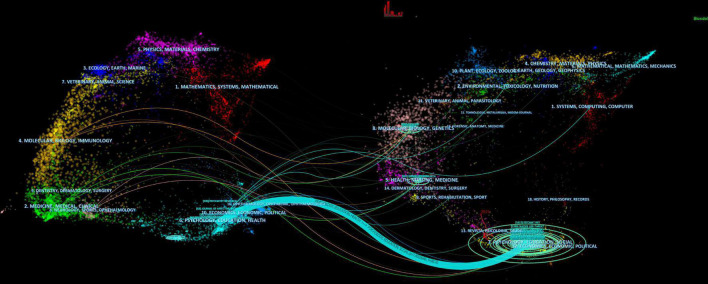
The dual-map overlay of journals contributed to publications on non-suicidal self-injury (NSSI) by CiteSpace. The left side represents the citing journals and the right side represents the cited journals.

The dual-map overlay of journals is shown in Figure 7. The left side represents the citing journals and the right side represents the cited journals. Those colored lines start from the left and end at the right presenting a co-citation relationship. From the figure, we can found that one main citation path. The publications related to NSSI were published in the journals of psychology, education, and health, but the most cited publications were published in the journals of psychology, education, and social.

### 3.5. Analysis of authors and cited authors

**FIGURE 8:**
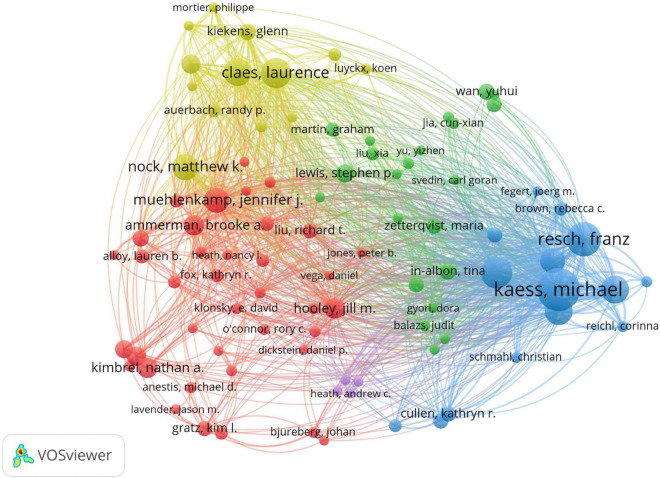
Network visualization map of authors generated by VOSviewer. Each node represents an author and each color represents one cluster, the links between nodes represents cooperation of authors.

**FIGURE 9:**
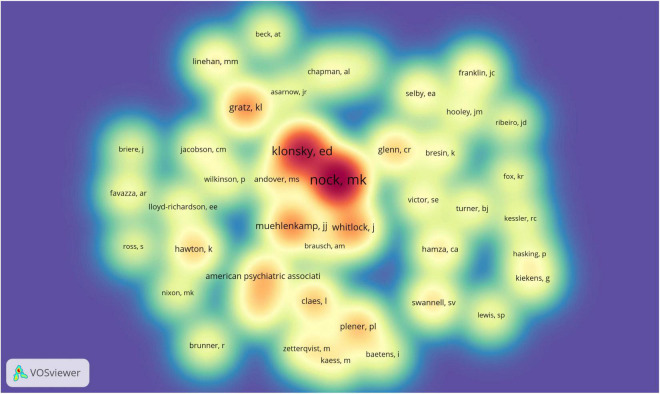
The density map of co-citation authors generated by VOSviewer. The deeper of color, the more representative authors is.

A total of 445 authors published related articles on NSSI. As shown in Figure 8, the author’s authorship was conducted by VOSviewer. Each color represents a cluster, there were 21 clusters in figure. Active collaborations usually exist in the same cluster, such as Michael Kaess and Peter Parzer; there were also collaborations among linked two nodes in different clusters, such as Matthew K. Nock and Laurence Claes (25). Besides, some clusters has no collaboration with others, which indicated further cooperation with authors in the field of NSSI. The top 10 related authors and co-citation are listed in Table 5. The most productive author was Michael Kaess, who contributed 35 publications, followed by Franz Resch (*n* = 26) and Paul L. Plener (*n* = 23). Co-cited author analysis refers to when the literatures of two authors are simultaneously cited by a third author. The visualization density map of co-citation author’s analysis was generated by VOSviewer (Figure 9), in which 43 authors were cited at least 100 times. We could clearly view the high-frequency co-cited authors, the deeper the color, the more citations. The highest frequency co-citation author is Matthew K. Nock, followed by E. David Klonsky and Jennifer J. Muehlenkamp.

**TABLE 5:** The top 10 authors and cited authors of non-suicidal self-injury (NSSI).

Ranking	Publications	Centrality	Author	Cited author	Citations
1	35	0.03	Michael Kaess	Matthew K. Nock	1412
2	26	0	Franz Resch	David Klonsky	989
3	23	0.07	Paul L. Plener	Jennifer J. Muehlenkamp	436
4	22	0.09	Laurence Claes	Kim L. Gratz	431
5	20	0	Peter Parzer	Janis Whitlock	414
6	20	0.01	Julian Koenig	American Psychiatric Association	303
7	20	0.02	Penelope Hasking	Laurence Claes	297
8	18	0.32	Matthew K. Nock	Paul L. Plener	296
9	17	0	Romuald Brunner	Catherine Rose Glenn	279
10	17	0.11	Jennifer J. Muehlenkamp	Keith Hawton	277

### 3.6. Analysis of co-cited references

**FIGURE 10:**
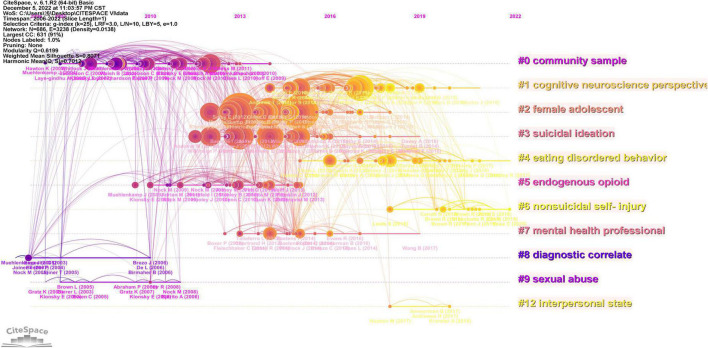
The network of co-cited references timeline viewer by CiteSpace. Each horizontal axis is one cluster, the denser the clusters are, the more they represent the frontier in the field of non-suicidal self-injury (NSSI).

Co-cited references are references that have been co-cited in a set of publications (26, 27). Usually cited references with the highest frequency are the main focus of the researchers. The top 10 co-cited references related to NISS are listed in Table 6, and each of them was co-cited more than 90 times. The first co-cited reference was published by Swannell SV et al., which investigated the methodological factors affecting heterogeneity in estimates of prevalence of NSSI, the time effects, and overall international NSSI prevalence (28). The second co-cited reference was published by Matthew K. Nock et al. In 2006, which reported on the diagnostic correlates of adolescents with a recent history of NSSI and examines the relation between NSSI and suicide attempts (29). The third co-cited reference was published by Matthew K. Nock et al. in 2004, which this study applied a functional approach to the assessment of self-mutilative behavior among adolescent psychiatric inpatients (30). The network map of co-cited references timeline view was conducted by CiteSpace (Figure 10), which displays the evolution of research hotspots over time. Clusters were formed by selecting keywords, and then a total of 11 clusters were generated by LLR algorithm; each of these clusters represents the activity of its future direction, the denser and more active the clusters in the graph, the more they represent the current research frontier (31). The elements on the horizontal axis represent co-cited references; the position of the element on the horizontal axis denotes the time of the first occurrence; and the line connecting the elements denotes the co-cited relationship. The size of the element is proportional to the citation count of the reference (32). As shown in Figure 10, cluster #0 (community sample), #8 (diagnostic correlate) and #9 (sexual abuse) started earlier; while cluster #4 (eating disordered behavior), and #6 (non-suicidal self-injury) are still continuous, which could be regarded as the frontier.

**TABLE 6:** The top 10 co-cited references of non-suicidal self-injury (NSSI).

Ranking	Co-cited reference	Frequency
1	Swannell et al. (28), suicide life-threat, V44, P273, DOI 10.1111/sltb.12070	233
2	Nock et al. (29), psychiat res, V144, P65, DOI 10.1016/j.psychres.2006.05.010	227
3	Nock and Prinstein (30), j consult clin psych, V72, P885, DOI 10.1037/0022-006x.72.5.885	189
4	Nock (52), annu rev clin psycho, V6, P399, DOI 10.1146/annurev.clinpsy.121208.131258	181
5	Klonsky (53), clin psychol rev, V27, P226, DOI 10.1016/j.cpr.2006.08.002	178
6	American Psychiatric Association (54), diagn stat man ment, V5th, DOI 10.1176/appi.books.9780890425596	161
7	Muehlenkamp et al. (55), child adolesc psychiatry ment health, V6, P10, DOI 10.1186/1753-2000-6-10	159
8	Lloyd-Richardson et al. (56), psychol med, V37, P1183, DOI 10.1017/s003329170700027x	143
9	Nock (57), curr dir psychol sci, V18, P78, DOI 10.1111/j.1467-8721.2009.01613.x	130
10	Nock et al. (58), psychol assessment, V19, P309, DOI 10.1037/1040-3590.19.3.309	122

**FIGURE 11:**
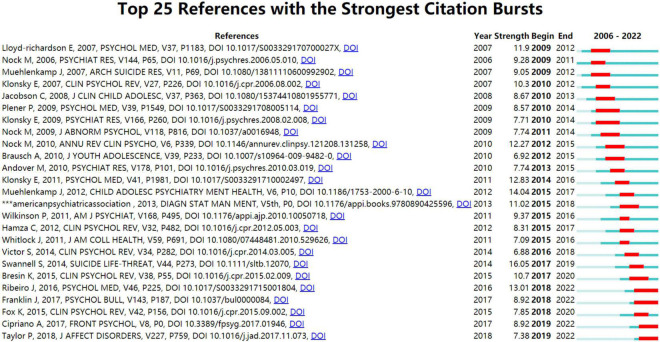
Top 25 references with the strongest citation bursts generated by CiteSpace. The blue line is the time interval, the red line is the time period with reference bursting.

References with citation bursts are those that have been cited significantly more frequently over a period (33). The years between “Begin” and “End” represent the period when the reference was more influential; years in light green mean that the reference has not yet appeared, years in dark green mean that the reference is less influential, and years in red mean that the reference is more influential (34). The top 25 references with the strongest citation bursts were generated by CiteSpace (Figure 11). The strongest burstness (strength = 27.35) occurred in a paper entitled “Prevalence of Non-suicidal Self-Injury in Non-clinical Samples: Systematic Review, Meta-Analysis and Meta-Regression”(28), published in Suicide Life Threat Behave by Sarah V Swannell et al. in 2014 with citation burstness from 2016 to 2019. Notably, five references (4, 5, 8, 35, 36) were still in burstness. Respectively, J D Ribeiro et al. (35) aimed to examine the magnitude and clinical utility of the associations between self-injurious thoughts and behaviors and subsequent suicide ideation, attempts, and death; G. Kiekens et al. (36) aimed to evaluate whether NSSI is associated with increased odds of subsequent onsets of suicidal thoughts and behaviors (STB) independent of common mental disorders, whether NSSI is associated with increased risk of transitioning from suicide ideation to attempt, and which NSSI characteristics are associated with STB after NSSI; Rebecca C. Brown et al. (8) reviewed the current literature on epidemiology, etiology, and therapeutic approaches with a focus on the period of adolescence; Peter J. Taylor et al. (4) conducted a systematic review and meta-analysis of the prevalence of NSSI functions in community and clinical samples; Annarosa Cipriano (5) reviewed non-suicidal, self-injurious behaviors.

### 3.7. Analysis of keywords

**FIGURE 12:**
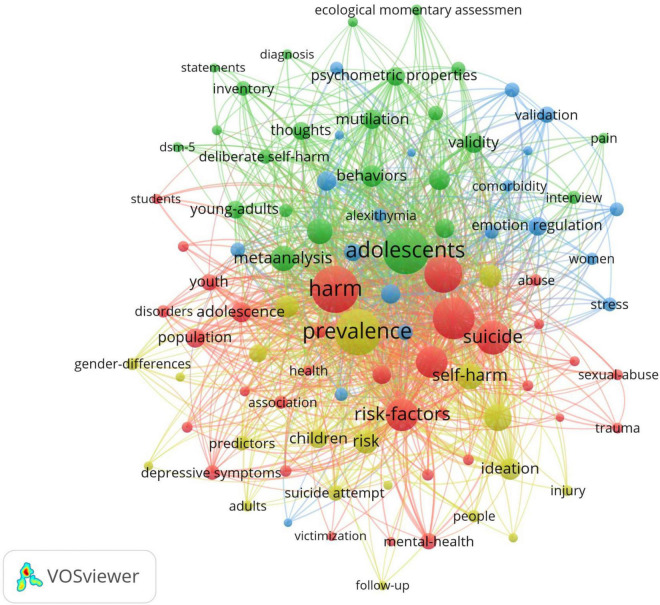
Keywords cluster analysis co-occurrence map generated by VOSviewer. Each node represents a keywords and each color represents one cluster, the links between nodes represents c0-occurrence of keywords.

**FIGURE 13:**
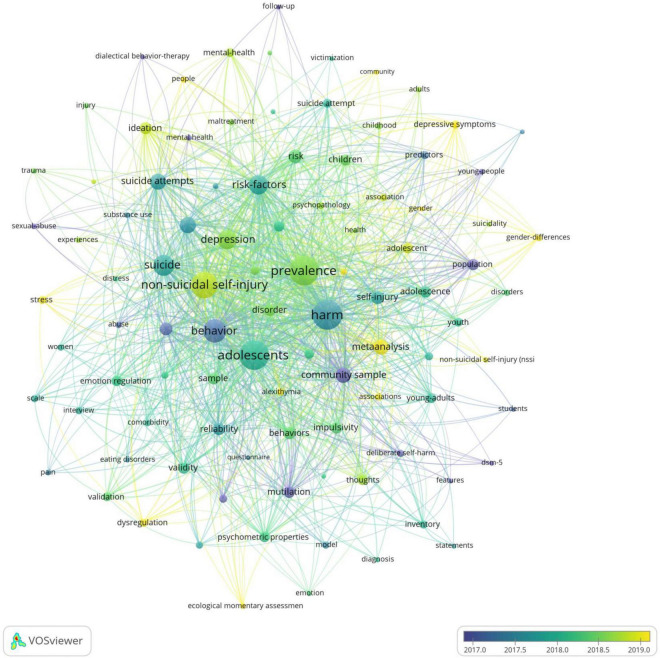
The overlay visualization of keywords generated by VOSviewer. The color of the nodes and lines indicated corresponding average appearing year of keywords.

A total of 484 keywords were extracted from literature about NSSI. 10 keywords were listed in Table 7. The most frequency was “harm” (*n* = 332), followed by “adolescents” (*n* = 323), “prevalence” (*n* = 321), “non-suicidal self-injury” (*n* = 267) and “behavior” (*n* = 220). The network of co-occurrence keywords was generated by VOSviewer (Figure 12), in which 97 keywords occurrence at 15 times. The keywords formed four clusters: cluster 1 (red color), cluster 2 (blue color), cluster 3 (yellow color), cluster 4 (green color), which were differentiated by color in the diagram, with the same color being the same cluster; the keyword size indicated the number of occurrences of the keyword, whereas the thickness and distance of the connecting lines between the keywords indicated the frequency of co-occurrence between the two keywords (37). The overlay visualization of keywords was generated by VOSviewer (Figure 13), where the color indicated the average published year. As we can see, ecological momentary assessment, dysregulation and gender difference are emerging fields that were colored yellow (17).

**TABLE 7:** Top 10 keywords in terms of frequency of non-suicidal self-injury (NSSI).

Ranking	Keywords	Frequency
1	harm	332
2	adolescents	323
3	prevalence	321
4	non-suicidal self-injury	267
5	behavior	220
6	suicide	178
7	risk-factors	163
8	depression	157
9	suicide attempt	115
10	community sample	106

**FIGURE 14:**
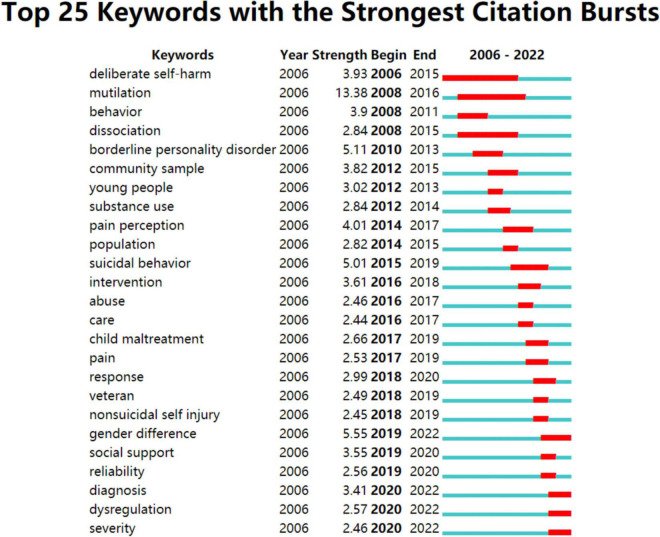
Top 25 keywords with the strongest citation bursts generated by CiteSpace. The blue line is the time interval, the red line is the time period with a keyword bursting.

The burst detection in Citespace, based on Kleinberg’s algorithm, aims to figure out a meaningful document flow structure with respect to time lapse. Keywords bursts provide helpful insight into the research footprint of the research focus (38). The top 25 keywords with the strongest citation bursts were generated by CiteSpace (Figure 14). The blue line means the time interval, while the red line means the time period with a keyword bursting (26). “mutilation” had the strongest burst (strength = 13.39) from 2008 to 2016. Notably, “gender difference” (strength = 5.56), “diagnosis”(strength = 3.41), “dysregulation” (strength = 2.58) are still in burstness, they are considered as frontier in the field of NSSI research.

## 4. Discussion

In this article, we used CiteSpace and VOSviewer to visually analyze studies on NSSI from 2002 to 2022, which aimed to help researchers grasp developmental trends in this field. 799 publications related to NSSI were searched from the WoSSC.

• As shown in Figure 2, the number of publications related to NSSI shows slightly fluctuated growth. There were small numbers of publications in this field that began to appear from 2002 to 2010, but in the meantime, however, this research is still in its infancy (28). Since 2011, the number of publications began to rapidly increase, it can be seen that the NSSI research is got more attention. Overall, the number of publications is up which represents NSSI research will has good development trend in the future.

According to the analysis of countries in Figure 5, the USA was the most productive country and the number of publications overwhelmingly exceeded other countries. It is inseparable from the local funding agencies (27). Among the top 10 funding agencies, five were from the USA and it shows the strong academic impact of the USA on the research related to NSSI. As shown in Table 1, five institutions were from the USA, and Harvard University shows the highest centrality (0.34), which indicates it plays a pivotal part in bridging cooperation among institutions worldwide.

Furthermore, according to the top 10 authors in Table 5, Michael Kaess from the Heidelberg University contributed the most publications. But Mattew K. Nock had the most value of centrality (0.32), which shows he played a vital leadership role in this field and it is worthwhile for novice researchers in this field to deeply research his publications. As for co-cited authors, the publications of five authors were cited more than 400 times and Matthew K. Nock (*n* = 1,412) had the highest number of citations. Significantly, Michael Kaess had the most significant number of published articles without ranking in top 10 cited journals, whereas Mattew K. Nock published articles far lwss than Michael Kaess could listed first in co-citation journals. Therefore, it can be seen that we should not only pay attention to the number of articles, but also the quality of articles, and improve the impact of the research in the future.

As shown in Table 4, the journal with the most articles about NSSI was the Psychiatry Research (*n* = 105, IF = 11.225) and there is only two journal’s impact factor exceeded 10.0, whereas there was a journal’s impact factor is 1.899 that JQR ranked Q4. It can be seen that publishing articles related to NSSI in high-IF journals are a huge challenge. As for co-cited journals, Psychiatry Research (*n* = 1,626) is also the journal with the highest citations. It is helpful to find the core journals published in the research about NSSI by analyzing the distribution of literature sources. It follows that the cited publications are all from high-impact journals, showing it is attached great importance to research NSSI in the worldwide academic field. The most co-cited reference was retrospective analysis of the prevalence of NSSI in non-clinical samples by Sarah V Swannell et al. (28).

The research hotspots and development dynamics in the field of NSSI can be captured from the keywords’ co-occurrence network and bursts reflect (39). By analyzing the frequency of the keywords and the keyword clustering analysis, it can be concluded that the research hotspots of NSSI are as follows:

(1) Study on the behavior character of NSSI and its harm to patients can help to find the corresponding treatment fundamentally (40).
(2) Study on the prevalence and risk factors of NSSI can better understand the phenomenology of NSSI and solve the different problems of the population (41). And the development of standardized methodology in NSSI research is vital when researchers estimate prevalence.
(3) Adolescent is the main group of NSSI research. The main cause is that numerous research showed that the NSSI prevalence of adolescents is higher. Surely, it also involved patients in the population of children (26), adults, bisexuality (27), and so on. The research population was gradually diversified and enriched.
(4) NSSI related to depression closely. Lu Wang, et al. (42) concluded that there is a relationship between NSSI and suicide risk in patients with depression. Koray Kara, et al. (43) discussed the relationship between NSSI and depression, concluded that depressive symptoms are more common in individuals with NSSI behaviors in a forensic adolescent population.

Additionally, keywords with the strongest citation bursts can reflect emerging trends and research frontiers. In this study, three frontiers of related research were captured as follows *via* CiteSpace: a meta-analysis (2018–2022), ecological momentary assessment (2020–2022), and diagnosis (2020–2022).

1. Gender difference: Gender difference in NSSI is not a static gap, but evolves across time, widening in mid-adolescence and disappearing by early adulthood (44). Moye Xin, et al. (45) investigated potential gender differences in the interrelations between different types of stressful life events and non-suicidal self injury (NSSI) among Chinese youth, clarifying the risk factors in affecting NSSI from male and female perspectives respectively. Fang Cheng, et al. (46) in their study found that in the path analysis model with the introduction of mediating effects, the influence of gender differences on NSSI behavior becomes more pronounced under the interaction of multiple factors: women seem to be more significantly influenced by the external derivatives in the internal derivatives than male subjects, and are more likely to trigger NSSI behavior under the interaction of multiple factors.
2. Diagnosis: Numerous research focus on studying diagnostic methods of the NSSI to promote to treat this disease. Maria Zetterqvist et al. (47) discussed the clinical utility of the NSSI diagnosis by using the Clinical Assessment of Non-suicidal Self-Injury Disorder Index (CANDI) and proved CANDI was a feasible tool to evaluate NSSI in adolescents not only restricted in adults. Jill M Hooley et al. (48) particularly analyze the NSSI diagnostic challenges and consider current issues in its diagnosis.
3. Dysregulation: Desregulation are usually considered as emotion dysregulation in the field of NSSI research, which many researchers find it closely connects with NSSI. Hedvig Andersson, et al. (49) considered that emotion dysregulation has been identified as a core mechanism in the development and maintenance of NSSI and it is therefore an important target when addressing NSSI. Additionally, So Yung Yang, et al. (50) also found the emotion dysregulation mediated the association between childhood trauma and NSSI and emphasize that promoting emotion regulation strategies could prevent NSSI behavior in patients with mood disorders.

## 5. Strengths and limitations

As far as we know, this is the first study in the Web of Science database that using bibliometric analysis to visually analyze NSSI from institutions, countries, authors, journals, co-cited references. Based on comprehensive indexes like journals, authors, countries, and institutions, bibliometric can conduct an in-depth evaluation of the research trends and the focus of a certain field; the combination of the knowledge map and visualized analysis can quantitatively reflect research status and practical applications simultaneously, and demonstrate the distribution of collaborations among countries, regions, disciplines, etc., (22, 51), other review types like systematic review and meta-analysis can’t make a summary from a monolithic and comprehensive perspective for specific fields of NSSI research. However, there also are some limitations to our study. The data about NSSI publications were only extracted from the WoSCC database and language restricted in English, which led to the data may not be comprehensive enough. Additionally, this study contains articles and reviews, and the credibility of the visual analysis may be reduced because of the uneven quality of the publications.

## 6. Conclusion

From bibliometric analysis and visualization of NSSI, it is can be seen that related literature developed rapidly at present. The USA is the most productive country and Michael Kaess is the most prolific author in this field. Psychiatry Research has the largest publications about NSSI. The research of NSSI hotspots and frontiers has been mentioned above and it will be very helpful to the researchers in this field.

## Data availability statement

The original contributions presented in this study are included in the article/supplementary material, further inquiries can be directed to the corresponding authors.

## Author contributions

WS and MH designed the research subject and critically revised the manuscript. YZ and GL conducted the literature retrieval and screening. XD, QZ, and SL provided the guidance in statistical analysis. XD, NZ, YZ, and GL wrote the manuscript. All authors read and approved the final manuscript.
